# Exposure to Bisphenol a Substitutes and Gestational Diabetes Mellitus: A Prospective Cohort Study in China

**DOI:** 10.3389/fendo.2019.00262

**Published:** 2019-04-30

**Authors:** Wenxin Zhang, Wei Xia, Wenyu Liu, Xinping Li, Jie Hu, Bin Zhang, Shunqing Xu, Yanqiu Zhou, Jiufeng Li, Zongwei Cai, Yuanyuan Li

**Affiliations:** ^1^Key Laboratory of Environment and Health (HUST), Ministry of Education and Ministry of Environmental Protection, State Key Laboratory of Environmental Health (Incubation), School of Public Health, Tongji Medical College, Huazhong University of Science and Technology, Wuhan, China; ^2^Wuhan Children's Hospital (Wuhan Maternal and Child Healthcare Hospital), Tongji Medical College, Huazhong University of Science and Technology, Wuhan, China; ^3^State Key Laboratory of Environmental and Biological Analysis, Department of Chemistry, Hong Kong Baptist University, Hong Kong, China

**Keywords:** gestational diabetes, bisphenol A, bisphenol S, bisphenol F, bisphenol AF, plasma glucose, endocrine disrupting chemicals

## Abstract

**Background:** The association of bisphenol A (BPA) and gestational diabetes mellitus (GDM) has been investigated in only a small number of studies, and research on the associations between BPA substitutes and GDM is scarce.

**Objective:** We aimed to investigate the associations of four bisphenols [bisphenol A (BPA), bisphenol S (BPS), bisphenol F (BPF), and bisphenol AF (BPAF)] levels in urine sample with the risk of gestational diabetes mellitus (GDM) and plasma glucose levels.

**Methods:** A total of 1,841 pregnant women from a cohort study were recruited at their first prenatal examination between 2013 and 2015 in Wuhan, China. Concentrations of four bisphenols (BPA, BPS, BPF, BPAF) were measured in first-trimester urine samples using Ultra-high performance liquid chromatography system coupled to a Triple Quadrupole mass spectrometer (UHPLC-TQMS). An oral glucose tolerance test (OGTT) was performed at 24–28 gestational weeks and GDM was diagnosed *post hoc* using International Association of Diabetes and Pregnancy Study Groups criteria. We used multivariable logistic regression models to examine the associations of urinary bisphenols with the risk of GDM, and multiple linear regression models to determine the associations between bisphenols exposure and plasma glucose levels.

**Results:** Urinary BPAF was associated with increased odds of GDM among women with normal pre-pregnancy BMI [adjusted odds ratio (aOR) = 1.70 (95% CI: 1.08, 2.67) for the highest group compared to the lowest group], and the association remained significant after additional adjustment for other bisphenols [aOR = 1.68 (95% CI: 1.03, 2.72)]. No significant associations were observed for other bisphenols and GDM. Consistent with the result of GDM, women in the highest BPAF category had a mean of 0.05 mmol/L (95% CI: 0.01, 0.09) higher fasting plasma glucose (FPG) levels than women in the lowest category. For BPA and plasma glucose, non-linear associations were observed between urinary BPA and FPG and the sum of the PG *z*-score among women who were overweight (*p* for non-linear association < 0.05). We also found that the per-unit increase in natural log transformed specific gravity adjusted BPS [ln (SG-adj BPS)] was associated with a 0.03 mmol/L (95% CI: 0.01, 0.04) increase in FPG levels and the associations might be modified by fetal sex (*p* for interaction < 0.05). Among women with female fetus, a per-unit increase in ln (SG-adj BPS) was associated with a 0.04 mmol/L (95% CI: 0.02, 0.06) increase in FPG, a 0.11 mmol/L (95% CI: 0.04, 0.17) increase in 1 h-PG and a 0.19 mmol/L (95% CI: 0.08, 0.30) increase in the sum of PG *z*-score.

**Conclusions:** Our results provide evidence that BPAF and BPS might be potential risk factors of GDM, which require to be studied further.

## Introduction

Gestational diabetes mellitus (GDM) is a common complication during pregnancy and is defined as “any degree of glucose intolerance with onset or first recognition during pregnancy” (1, 2). GDM and hyperglycemia during pregnancy have been reported to be associated with adverse maternal, neonatal, and postnatal outcomes; thus, it is important to find potential risk factors for GDM. Except for the common-known risk factors (a high maternal age, being overweight before pregnancy, a family history of type 2 diabetes, a history of diabetes before pregnancy, etc.), concerns are increasingly being raised on the environmental factors for developing GDM, especially for some environmental chemicals that have endocrine-disrupting effects (3–5).

Bisphenol A (BPA, 2,2-bis(4-hydroxyphenyl)-propane), a typical endocrine disruptor, is widely used in the production of polycarbonate plastics and epoxy resins used in numerous consumer products, to which human beings are widely exposed to in daily life. Increasing evidence has indicated that BPA may be harmful to human health, especially with regard to endocrine metabolism (6–9). Evidence from animal studies has suggested that BPA exposure may disrupt glucose homeostasis and contribute to metabolic disorders; thus, BPA may be a risk factor for the development of diabetes (10–12). Epidemiological studies also suggested that BPA was associated with type 2 diabetes among the general population (13–16). In terms of pregnant women, as far as we were aware, only five epidemiological studies addressed the associations of urinary BPA concentration with blood glucose levels or GDM, but the conclusions were inconsistent (17–21).

Due to public concern on the potential harmful effects of BPA exposure, bisphenol analogs were used to substitute BPA in the manufacture of certain plastics and epoxy resins (22, 23). Three widespread and commercially used bisphenol analogs are bisphenol S (BPS, 4,4′-sulfonyldiphenol), bisphenol F (BPF, 4,4′-dihydroxydiphenylmethane), and bisphenol AF (BPAF, 2,2-bis(4-hydroxyphenyl)-hexafluoropropane) (23, 24). According to previous studies, BPA, BPS, BPF, and BPAF were detected in indoor dust samples and in food and beverages (25, 26), which indicates an ubiquitous exposure to bisphenols of human beings.

Considering the structural similarity to BPA, the analogs (BPS, BPF, BPAF) may have similar endocrine-disrupting effects to those of BPA (23, 27, 28). Studies in zebrafish have reported the disrupting effects on steroid hormones of BPS (29), BPF (30), and BPAF (31). However, the potential harmful effects on glucose homeostasis of these BPA substitutes, which were used more and more widely and frequently in our daily life, are still unknown.

Since findings on the associations between BPA exposure and the risk of GDM were inconsistent, and little was known on the endocrine-disrupting effects on human metabolism of BPA substitutes, we conducted the prospective cohort study to investigate the potential disrupting effects of bisphenols exposure on glucose metabolism. In this prospective study, we examined concentrations of four typical and widely used bisphenols (BPA, BPS, BPF, BPAF) in first-trimester urine samples of pregnant women and estimated the associations of the four bisphenols with GDM and plasma glucose levels in a population of pregnant women in central China. Meanwhile, it was reported that women with a different BMI before pregnancy or who were carrying a fetus of a different sex have different endocrine environments (32–34), so we carried out a further stratified analysis by pre-pregnancy BMI and fetal sex.

## Methods

### Study Population

We conducted this prospective study and recruited pregnant women at the Wuhan Maternal and Child Healthcare Hospital (WMCHH) in Wuhan, China. A total of 2,145 eligible pregnant women with donated urine samples were recruited at WMCHH between October 2013 and April 2015. Eligibility criteria included singleton pregnancy, gestational age < 16 weeks at enrollment, and a willingness to give birth at the study hospital.

Among 2,145 pregnant women, we first excluded those who did not undertake the GDM screening during pregnancy (*n* = 301). Women with reported a family history of diabetes or reported a history of diabetes before pregnancy were also excluded (*n* = 3). Eventually, 1,841 pregnant women were included in the final analysis.

This study was approved by the ethics committees of Tongji Medical College, Huazhong University of Science and Technology [No. (2012)07], and Wuhan Maternal and Child Healthcare Hospital (No. 2012003). All participants agreed and signed informed consent.

### Urine Samples Collection and Bisphenols Measurements

Urine samples were collected at 13 wk of gestation on average (ranging from 8 to 18 wk), and stored in polypropylene containers at −20°C until further analysis.

Urinary bisphenols concentrations were quantified using an Ultimate 3000 Ultra-high performance liquid chromatography system (Dionex, Sunnyvale, CA, USA) coupled to a Thermo Scientific™ TSQ Quantiva™ Triple Quadrupole mass spectrometer (Thermo Scientific, San Jose, CA) (UHPLC-TQMS) with the isotope labeled internal standards purchased from Sigma-Aldrich (St. Louis, U.S.A), which was described in our previous study (35). Briefly, 1 mL of urine sample was incubated with β-glucuronidase at 37°C overnight mixed with the internal standard solution (with the final concentration of 20 ng/mL). After enzymatic hydrolysis, the solution was extracted 3 times with a 3 mL solvent [methyl tert-butyl ether/ethyl acetate (5/1, v/v)] each time. The supernatants were combined and evaporated under nitrogen gas flow, and then reconstituted in 200 μL acetonitrile/water (6/4, v/v). Chromatographic separation was achieved on Thermo Scientific Betasil C18 column (2.1 mm × 100 mm, 3 μm) using a mobile phase gradient with water and acetonitrile. The compounds were detected by negative-ion electrospray ionization mass spectrometry and multiple reaction monitoring mode. The blanks and quality control samples were incorporated into each batch of samples. As reported before, the limit of detection (LOD), defined as the concentrations producing a signal-to-noise ratio equal to 3, was 0.2 μg/L for BPA and BPS, 0.1 μg/L for BPF and BPAF (35).

Considering the individual variation of urine dilution, we adjusted the bisphenols concentration by urine specific gravity (SG), which was measured by a handheld digital refractometer (Atago, Tokyo, Japan). The following formula was used to adjust urinary concentrations of bisphenols:

SG-adjusted Bisphenols (μg/L) = unadjusted Bisphenols (μg/L) × [(SG_m_-1)/(SG_i_-1)]

where SG_m_ is the median SG (SG_m_ = 1.014) of all the samples (*n* = 1,841), and SG_i_ is the observed SG for the individual urine sample.

### GDM Diagnosis and Plasma Glucose Measurements

All pregnant women were routinely required to undertake the one-step GDM screening—a 2 h 75 g oral glucose tolerance test (OGTT) at 24–28 weeks of gestation in the study hospital. The diagnosis of GDM was according to the International Association of the Diabetes and Pregnancy Study Groups (IADPSG) criteria: fasting plasma glucose (FPG) ≥ 5.1 mmol/L (≥ 92 mg/dL), or 1 h plasma glucose (1 h-PG) ≥ 10.0 mmol/L (≥ 180 mg/dL), or 2 h plasma glucose (2 h-PG) ≥ 8.5 mmol/L (≥ 153 mg/dL) (2). We extracted glucose laboratory data of OGTT from the medical information system of the hospital, which recorded FPG (*n* = 1,841), 1 h-PG (*n* = 1,830), 2 h-PG (*n* = 1826) measured values for pregnant women in this study.

### Covariates

For each participant, a face-to-face interview was conducted within 3 days before or after delivery by specially trained nurses to collect a variety of information, including demographic and socioeconomic characteristics (e.g., maternal age, occupation, and education levels) and lifestyle factors during pregnancy (e.g., smoking, passive smoking, and alcohol consumption). Pre-pregnancy body mass index (BMI, kg/m^2^) was calculated using self-reported pre-pregnancy weight, which was extracted from the records of the first prenatal visit, and height was measured at the hospital. Information on family history of diabetes, diabetes history, and the infant's sex were retrieved from the medical information system mentioned above.

### Statistical Analysis

Descriptive statistics were conducted to summarize the characteristics of the GDM group and non-GDM group in our study population. For measured bisphenol concentrations below the LOD, we assigned a value equal to the LOD divided by the square root of 2 in the analysis (36). Due to the skewed distribution of SG-adjusted bisphenols concentrations, we used the natural log-transformed values for further analysis, and the natural log-transformed SG-adjusted BPA concentration was abbreviated as ln (SG-adj BPA). We performed a Spearman correlation analysis to assess the correlations between urinary bisphenols [ln (SG-adj bisphenols)].

We selected the covariates included in the final models based on either their biologic plausibility (regardless of statistical significance) or the association with GDM in bivariate analysis (*p* < 0.10). According to these criteria, pre-pregnancy BMI (< 18.5 kg/m^2^, 18.5–23.0 kg/m^2^, ≥ 23 kg/m^2^), maternal age at delivery (years) and educational levels (high school or lower, college, university or above) were selected based on bivariate analysis (*p* < 0.10), and parity (nulliparous, multiparous), passive smoking during pregnancy (yes, no) and fetal sex (male, female) were selected based on biologic plausibility reported by previous studies.

We categorized participants into tertiles based on the distribution of SG-adjusted urinary BPA, BPS, and BPF concentrations, and BPAF concentration was categorized into a binary variable using the 66.6th percentile as the cut-point because 1,057 (57.41%) objects have a concentration value lower than LOD. We first used the multivariable logistic regression model to assess the association of bisphenols levels and GDM. Two multivariable regression models were conducted—model 1 was designed to investigate the effect of single bisphenol exposure and odds ratios for GDM with an adjustment for the covariates mentioned above, and model 2 was aimed to explore the co-exposure effects of multi-bisphenols by considering other bisphenols additionally in one model. We further conducted stratified analysis of bisphenols and GDM among women with normal weight (18.5 kg/m^2^ q pre-pregnancy BMI < 22.9 kg/m^2^) and women who were overweight (23.0 kg/m^2^ q pre-pregnancy BMI < 28.0 kg/m^2^). We selected the BMI cut-off point of 23.0 kg/m^2^ for overweight subjects, according to a reported optimal cut-off value of BMI for urban Chinese female adults (37). A BMI of 28.0 kg/m^2^ was used to discriminate between overweight and obesity according to the Working Group on Obesity in China (WGOC). Since few had met the criteria of being obese in our population (*n* = 41) and considering the potential confounding effects of a disrupted endocrine environment due to extreme body weight status, we excluded those women with obesity in the BMI-stratified analysis.

To examine the associations between urinary bisphenol concentrations and plasma glucose (PG) levels, we performed multiple linear regression models for the continuous variables of glucose measurements, and bisphenol concentrations were treated as categorical variables and continuous variables, respectively. For continuous variables of bisphenols, we calculated the results with per-unit increases in ln (SG-adj bisphenols). We calculated *z*-scores for FPG, 1 h-PG, and 2 h-PG by subtracting the mean from each woman's glucose measurement in this study and dividing it by the corresponding standard deviation; the sum of the three resulting *z*-scores for each woman was used as an outcome variable (38, 39).

Pre-pregnancy BMI and the fetal sex were evaluated as potential effect modifiers, and stratified analyses were performed. In the BMI-stratified analysis, we restricted our analysis to the women with normal and overweight BMI group. We calculated the *p*-value for trend in analysis using the median values of each category of bisphenol and set it as a continuous variable in the statistical model. We calculated the *p*-value for interaction in the stratified analysis using likelihood ratio tests to examine the significance of interaction terms between continuous bisphenol concentration and the stratified variable. In the tertile analysis of BPA and glucose levels in overweight group, we observed that BPAs in the middle tertile were associated with decreased plasma glucose levels. To verify whether there were non-linear relationships between bisphenols and glucose levels or GDM, we conducted a restricted cubic spline (RCS) analysis for bisphenols among overweight participants.

All statistics were performed using SAS version 9.4 (SAS institute, Cary, NC). A two-sided *p* < 0.05 was considered as statistically significant.

## Results

The characteristics of 1,841 participants in this study are shown in Table 1. Among 1,841 participants, 167 (9.07%) women were diagnosed with GDM. Compared to women without GDM, women with GDM were older (30.07 vs. 28.44, years), had greater pre-pregnancy BMI (22.32 vs. 20.76, kg/m^2^), had lower educational levels (the proportion of high school or lower was 28.14% vs. 18.40%), and were more likely to be multiparous (18.56% vs. 11.65%). No significant differences were observed in passive smoking and fetal sex for women with GDM vs. non-GDM (Table 1).

**Table 1:** Characteristics of 1,841 participants in this study.

**Characteristics**	**All participants (*n* = 1,841)**	**GDM (*n* = 167)**	**Non-GDM (*n* = 1,674)**	***P*-Value^**a**^**
Maternal age (years)	28.58 ± 3.27	30.07 ± 4.11	28.44 ± 3.14	<0.01
Pre-pregnancy BMI (kg/m^2^)	20.90 ± 2.87	22.32 ± 3.03	20.76 ± 2.81	<0.01
Pre-pregnancy BMI categories (kg/m^2^)				<0.01
<18.5	336 (18.25)	12 (7.19)	324 (19.35)	
18.5–22.99	1,162 (63.12)	84 (50.30)	1,078 (64.40)	
≥23	343 (18.63)	71 (42.51)	272 (16.25)	
Maternal education				0.01
High school or lower	355 (19.28)	47 (28.14)	308 (18.40)	
Some college	560 (30.42)	50 (29.94)	510 (30.47)	
University or above	926 (50.30)	70 (41.92)	856 (51.14)	
Passive smoking during pregnancy				0.30
No	1,246 (67.68)	107 (64.07)	1,139 (68.04)	
Yes	595 (32.32)	60 (35.93)	535 (31.96)	
Parity				0.01
0	1,615 (87.72)	136 (81.44)	1,479 (88.35)	
≥1	226 (12.28)	31 (18.56)	195 (11.65)	
Fetal sex				0.57
Male	976 (53.01)	85 (50.90)	891 (53.23)	
Female	865 (46.99)	82 (49.10)	783 (46.77)	

*BMI, body mass index; GDM, gestational diabetes mellitus; Data were presented as N (%) and Mean ± SD*.

a *p-Values estimates were based on Student's t-test for continuous variables expressed as Mean ± SD, and Pearson chi-squared test for categorical variables expressed as N (%)*.

Table 2 shows the distributions of urinary bisphenols concentrations, and plasma glucose levels at 24–28 weeks of gestation. BPF had the highest detection rate (>LOD) (94.72%), followed by BPS (90.06%), BPA (79.25%), and BPAF (42.53%). Similarly, BPF had the highest geometric mean (GM) (1.74 μg/L for un-adjusted and 2.01 μg/L for SG-adjusted) and BPAF had the lowest GM (0.025 μg/L for un-adjusted and 0.030 μg/L for SG-adjusted). The high detection rates of urinary bisphenols concentration suggested that the participants in this study were widely and frequently exposed to BPA substitutes. Urinary bisphenols showed weakly pairwise correlations, with Spearman correlation coefficients lower than 0.3 (Table S1). The arithmetic mean (AM) of fasting plasma glucose (FPG) was 4.35 mmol/L, the AM of plasma glucose after 1 h (1 h-PG) was 6.99 mmol/L, and the AM of plasma glucose after 2 h (2 h-PG) was 6.31 mmol/L. Their median values and selected percentiles are presented in Table 2.

**Table 2:** Distributions of first-trimester urinary bisphenols concentrations and plasma glucose levels.

**Bisphenols**	***N***	**>LOD (%)**	**GM (95% CI) or AM ± SD**	**P25**	**P50**	**P75**	**P95**
**UN-ADJUSTED (μg/L)**							
BPA	1,841	79.25	0.72 (0.66, 0.79)	0.27	1.11	2.66	10.05
BPS	1,841	90.06	0.30 (0.28, 0.32)	0.12	0.25	0.64	4.43
BPAF	1,841	42.59	0.025 (0.024, 0.026)	<LOD	<LOD	0.033	0.13
BPF	7,76^a^	94.72	1.74 (1.51, 1.99)	0.60	1.28	8.05	42.14
**SG-ADJUSTED (μg/L)**							
BPA	1,841	79.25	0.87 (0.79, 0.96)	0.34	1.41	3.13	14.71
BPS	1,841	90.06	0.36 (0.33, 0.38)	0.14	0.31	0.81	5.51
BPAF	1,841	42.59	0.030 (0.028, 0.031)	<LOD	<LOD	0.049	0.21
BPF	776^a^	94.72	2.01 (1.75, 2.32)	0.60	1.74	8.72	44.70
**PLASMA GLUCOSE LEVELS (mmol/L)**							
FPG	1,841	100%	4.35 ± 0.48^d^	4.07	4.31	4.57	5.04
1 h-PG	1,830^b^	100%	6.99 ± 1.57^d^	5.89	6.83	7.93	9.80
2 h-PG	1,826^c^	100%	6.31 ± 1.29^d^	5.49	6.19	6.98	8.49
Sum of PG *z*-score	1,820^d^	NA	−0.01 ± 2.42^d^	−1.53	−0.29	1.10	3.97

*LOD, limit of detection; GM, geometric mean; AM, arithmetic mean; SD, standard deviation; SG, specific gravity; FPG, fasting plasma glucose; PG, plasma glucose; NA, not available*.

a *For BPF, 776 urine samples were examined*.

b *Among 1,841 subjects, 11 didn't have the 1 h-PG measures in medical records*.

c *Among 1,841 subjects, 15 didn't have the 2 h-PG measures in medical records*.

d *Among 1,841 subjects, 1,820 (98.86%) has complete 3 time points OGTT measures*.

Table 3 shows the associations of SG-adjusted urinary bisphenol concentrations in tertiles (BPAF was categorized to concentration ≥66.6 percentage (0.036 μg/L) or below) with GDM. We did not observe any significant associations between the urinary levels of bisphenols and GDM among all participants. However, in stratified analysis, the highest category of BPAF was significantly associated with increased odds of GDM among women with normal weight (18.5 ≤ BMI < 23 kg/m^2^, *n* = 1,162) compared to the lowest category [odds ratio (OR) = 1.70 (95% CI: 1.08, 2.67) after adjustment for maternal age, educational levels, parity, passive smoking, and fetal sex]. The association remained significant after further adjustments for urinary BPA and BPS levels. No significant associations of levels of BPA, BPS, and BPF were found with GDM (Table 3).

**Table 3:** Associations of urinary concentrations of bisphenols and GDM.

**Bisphenols**	**All participants (*****n*** **= 1,841)**			**18.5 q BMI < 23 kg/m**^****2****^ **(*****n*** **= 1,162)**			**23 q BMI < 28 kg/m**^****2****^ **(*****n*** **= 302)**		
	**GDM/Total**	**Model 1**	**Model 2**	**GDM/Total**	**Model 1^**a**^**	**Model 2^**a**^**	**GDM/Total**	**Model 1^**a**^**	**Model 2^**a**^**
		**OR (95% CI)**	**OR (95% CI)**		**OR (95% CI)**	**OR (95% CI)**		**OR (95% CI)**	**OR (95% CI)**
**BPA**									
Low	61/613	Reference	Reference	24/387	Reference	Reference	30103	Reference	Reference
Medium	51/613	0.84 (0.56, 1.26)	0.84 (0.56, 1.26)	26/381	1.14 (0.64, 2.03)	1.13 (0.63, 2.03)	18/105	0.53 (0.26, 1.06)	0.53 (0.26, 1.07)
High	55/615	0.94 (0.63, 1.40)	0.90 (0.60, 1.37)	34/394	1.38 (0.80, 2.39)	1.22 (0.69, 2.16)	18/94	0.59 (0.29, 1.19)	0.61 (0.28, 1.31)
*p* for trend		0.90	0.71		0.24	0.51		0.21	0.27
**BPS**									
Low	58/614	Reference	Reference	26/366	Reference	Reference	25/113	Reference	Reference
Medium	58/612	1.06 (0.71, 1.58)	1.07 (0.72, 1.60)	27/394	0.94 (0.53, 1.65)	0.92 (0.52, 1.62)	25/95	1.59 (0.81, 3.15)	1.66 (0.83, 3.30)
High	51/615	0.89 (0.59, 1.34)	0.84 (0.55, 1.28)	31/402	1.05 (0.61, 1.82)	0.88 (0.50, 1.55)	16/94	0.79 (0.38, 1.64)	0.83 (0.38, 1.81)
*p* for trend		0.45	0.30		0.76	0.70		0.26	0.37
**BPAF**									
Low	109/1,227	Reference	Reference	46/769	Reference	Reference	50/223	Reference	Reference
High	58/614	1.24 (0.87, 1.76)	1.33 (0.91, 1.93)	38/393	1.70 (1.08, 2.67)^*^	1.68 (1.03, 2.72)^*^	16/79	0.97 (0.49, 1.91)	1.22 (0.57, 2.60)
*p* for trend		0.23	0.14		0.02^*^	0.04^*^		0.93	0.61
**BPF (*****n*** **= 776)**^**b**^									
Low	21/258	Reference	Reference	9/164	Reference	Reference	10/38	Reference	Reference
Medium	24/258	1.24 (0.65, 2.36)	1.25 (0.65, 2.42)	14/158	1.67 (0.69, 4.09)	1.55 (0.62, 3.90)	7/40	0.73 (0.23, 2.29)	0.85 (0.25, 2.85)
High	30/260	1.29 (0.69, 2.41)	1.25 (0.66, 2.38)	17/157	2.06 (0.86, 4.93)	1.87 (0.75, 4.65)	12/65	0.70 (0.25, 1.96)	0.93 (0.31, 2.81)
*p* for trend		0.56	0.66		0.18	0.28		0.62	0.99

*BMI, body mass index; GDM, gestational diabetes mellitus. Model 1: Adjusted for maternal age, pre-pregnancy BMI, educational levels, parity, passive smoking and fetal sex. Model 2: Additionally adjusted for other bisphenols based on model 1, for BPA, BPS, BPAF, adjustments was except for BPF due to the differences in sample size*.

a *Adjusted for confounders above except for pre-pregnancy BMI*.

b *Urine samples of 776 participants were measured for BPF. Among them, 479 women's pre-pregnancy BMI were between 18.5 and 22.9, and 143 participants' pre-pregnancy BMI were between 23 and 27.9 (kg/m^2^)*.

* *Significant p-value*.

Table 4 presents the associations of urinary bisphenol levels and plasma glucose levels among all participants. Compared to the lowest category of BPAF, women in the highest category had a mean of 0.05 mmol/L (95% CI: 0.01, 0.09) higher fasting glucose concentration. We also observed that BPS was associated with increased FPG levels [β = 0.03 (95% CI: 0.01, 0.04)] and an increased sum of PG *z*-score [β = 0.07 (95% CI: −0.00, 0.14)] with per-unit increases in ln (SG-adj BPS). We further performed a stratified analysis to investigate the associations of urinary bisphenols with glucose levels among women with normal weight and overweight, respectively. The results showed that BPAF was significantly associated with increased FPG [β = 0.07 (95% CI: 0.02, 0.12)] and the sum of the PG *z*-score [β = 0.26 (95% CI: 0.01, 0.50)] for the highest category compared to the lowest one after adjustment for potential confounders among women with normal pre-pregnancy BMI (Table S2). BPA in the second tertile was found to be associated with decreased FPG [β = −0.19 (95% CI: −0.36, −0.02)] and 2 h-PG levels [β = −0.43 (95% CI: −0.84, −0.03)] and the sum of the PG *z*-score [β = −0.95 (95% CI: −1.77, −0.13)] compared to the first tertile among overweight group, but there was no significance in trend analysis (Table S3). No other significant associations of bisphenols and GDM or plasma glucose levels were observed in pre-pregnancy BMI and fetal sex stratified analysis (Tables S4, S5, and S7).

**Table 4:** Associations of urinary bisphenols and glucose levels among all participants (*n* = 1,841)^a^.

**Bisphenols**	**FPG**		**1 h-PG**		**2 h-PG**		**Sum of PG** ***z*****-scores**	
	**b (95% CI)**	***p***	**b (95% CI)**	***p***	**b (95% CI)**	***p***	**b (95% CI)**	***p***
**BPA**								
Per-unit increase in ln (SG-adj BPA)	0.00 (−0.01, 0.01)	0.95	−0.02 (−0.05, 0.02)	0.32	−0.01 (−0.04, 0.02)	0.43	−0.02 (−0.07, 0.03)	0.42
Low	Reference		Reference		Reference		Reference	
Medium	−0.04 (−0.09, 0.01)	0.12	−0.05 (−0.22, 0.11)	0.53	−0.01 (−0.14, 0.13)	0.94	−0.13 (−0.38, 0.13)	0.34
High	0.01 (−0.04, 0.06)	0.68	−0.05 (−0.22, 0.12)	0.55	0.00 (−0.14, 0.14)	0.99	−0.02 (−0.28, 0.24)	0.88
*p* for trend		0.39		0.62		0.98		0.94
**BPS**								
Per–unit increase in ln (SG–adj BPS)	0.03 (0.01, 0.04)	<0.01^*^	0.04 (−0.01, 0.09)	0.09	0.01 (−0.03, 0.05)	0.54	0.07 (−0.00, 0.14)	0.05
Low	Reference		Reference		Reference		Reference	
Medium	0.03 (−0.02, 0.09)	0.20	0.13 (−0.04, 0.30)	0.14	−0.03 (−0.17, 0.11)	0.70	0.12 (−0.14, 0.37)	0.37
High	0.05 (−0.00, 0.10)	0.06	0.14 (−0.03, 0.31)	0.10	0.01 (−0.12, 0.15)	0.84	0.18 (−0.07, 0.44)	0.16
*p* for trend		0.11		0.21		0.70		0.22
**BPAF**								
Per–unit increase in ln (SG–adj BPAF)	0.01 (−0.00, 0.03)	0.13	0.01 (−0.05, 0.08)	0.66	0.00 (−0.05, 0.05)	0.99	0.03 (−0.06, 0.12)	0.53
Low	Reference		Reference		Reference		Reference	
High	0.05 (0.01, 0.09)	0.03^*^	0.04 (−0.11, 0.18)	0.64	0.05 (−0.07, 0.17)	0.39	0.15 (−0.07, 0.37)	0.19
*p* for trend		0.03^*^		0.64		0.39		0.19
**BPF (*****n*** **= 776)**								
Per–unit increase in ln (SG-adj BPF)	0.01 (−0.01, 0.02)	0.51	0.02 (−0.03, 0.08)	0.45	−0.02 (−0.06, 0.03)	0.42	0.01 (−0.08, 0.09)	0.87
Low	Reference		Reference		Reference		Reference	
Medium	0.01 (−0.08, 0.09)	0.90	−0.08 (−0.35, 0.20)	0.58	−0.18 (−0.40, 0.04)	0.12	−0.20 (−0.60, 0.21)	0.34
High	0.01 (−0.08, 0.10)	0.89	−0.07 (−0.34, 0.21)	0.64	−0.20 (−0.42, 0.02)	0.08	−0.18 (−0.59, 0.23)	0.38
*p* for trend		0.93		0.80		0.22		0.60

*SG, specific gravity; FPG, fasting plasma glucose; PG, plasma glucose*.

a *Adjusted for maternal age, pre-pregnancy BMI, educational levels, parity, passive smoking and fetal sex*.

* *Significant p-value*.

**Figure 1:**
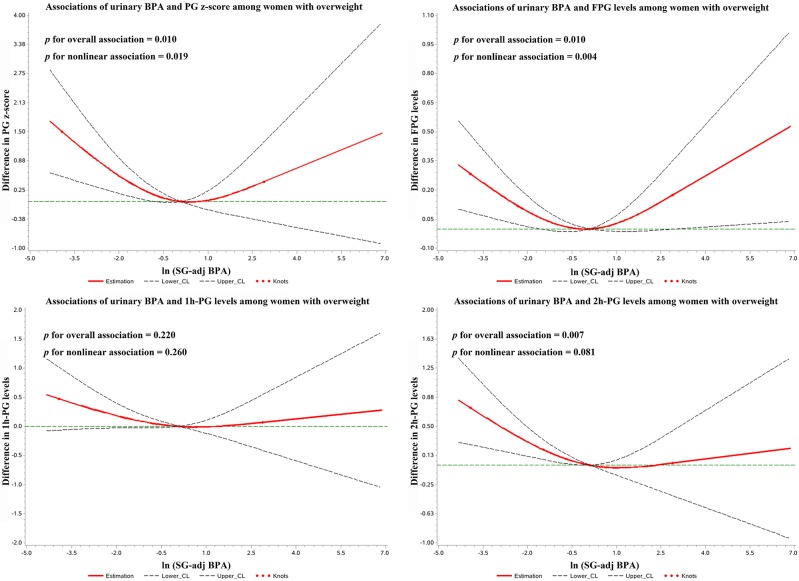
Restricted cubic spline for the associations between urinary BPA and plasma glucose levels among women with overweight. The red lines represent differences in glucose levels for natural log transformed specific gravity adjusted urinary BPA concentration with adjustment for maternal age, educational levels, parity, passive smoking, and fetal sex. Knots were set at the 5th, 50th, 95th percentiles and the reference value was set to median of urinary BPA distribution among women with overweight. Dashed lines represent 95% CI.

Figure 1 showed the restricted cubic spline analysis for the associations between BPA and glucose levels among women who were overweight. Non-linear associations were observed among overweight women in terms of fasting plasma glucose levels and *z*-score of plasma glucose (*p* for non-linear association < 0.05). The dose-response relationships between BPA and plasma glucose levels also indicated a “U-shaped” association between BPA exposure and fasting plasma glucose levels (Figure 1). However, no significant non-linear association was observed for GDM among overweight women (Figure S1).

Additionally, we found that the associations between BPS and plasma glucose might be modified by fetal sex (*p* for interaction < 0.01 for sum of PG *z*-score and 1 h-PG, < 0.05 for FPG) (Table 5). Specifically, significant results were only observed among women with female fetus and the per-unit increase in ln (SG-adj BPS) was associated with higher FPG [β = 0.04 (95% CI: 0.02, 0.06)], 1 h-PG [β = 0.11 (95% CI: 0.04, 0.17)], and the sum of the PG *z*-score [β = 0.19 (95% CI: 0.08, 0.30)].

**Table 5:** Associations of BPS concentrations with glucose levels, stratified by fetal sex^a^.

**BPS**	**FPG**		**1 h PG**		**2 h-PG**		**Sum of PG** ***z*****-scores**	
	**b (95% CI)**	***p***	**b (95% CI)**	***p***	**b (95% CI)**	***p***	**b (95% CI)**	***p***
**WOMEN WITH FEMALE FETUS (*****n*** **= 865)**								
Per-unit increase in ln (SG-adj BPS)	0.04 (0.02, 0.06)	<0.01^*^	0.11 (0.04, 0.17)	<0.01^*^	0.04 (−0.01, 0.10)	0.13	0.19 (0.08, 0.30)	<0.01^*^
Low	Reference		Reference		Reference		Reference	
Medium	0.04 (−0.04, 0.12)	0.31	0.07 (−0.18, 0.31)	0.60	0.00 (−0.21, 0.21)	1.00	0.11 (−0.27, 0.50)	0.56
High	0.11 (0.03, 0.19)	<0.01^*^	0.28 (0.04, 0.52)	0.02^*^	0.08 (−0.13, 0.28)	0.46	0.45 (0.07, 0.84)	0.02^*^
*p* for trend		<0.01^*^		0.02^*^		0.40		0.02^*^
**WOMEN WITH MALE FETUS (*****n*** **= 976)**								
Per-unit increase in ln (SG-adj BPS)	0.01 (−0.01, 0.03)	0.15	−0.01 (−0.08, 0.05)	0.66	−0.01 (−0.06, 0.04)	0.59	−0.03 (−0.12, 0.06)	0.56
Low	Reference		Reference		Reference		Reference	
Medium	0.03 (−0.04, 0.10)	0.41	0.20 (−0.03, 0.44)	0.09	−0.04 (−0.22, 0.15)	0.70	0.14 (−0.20, 0.48)	0.42
High	0.00 (−0.07, 0.07)	0.99	0.03 (−0.21, 0.26)	0.82	−0.05 (−0.23, 0.14)	0.63	−0.05 (−0.39, 0.29)	0.78
*p* for trend		0.73		0.66		0.7		0.51
*p* for interaction^b^		0.03^*^		<0.01^*^		0.11		<0.01^*^

*SG, specific gravity; FPG, fasting plasma glucose; PG, plasma glucose*.

a *Adjusted for maternal age, pre-pregnancy BMI, educational levels, parity, passive smoking*.

b p for interaction was calculated using the interaction term of ln (SG-adj BPS)*sex

* *Significant p-value*.

## Discussions

While the relationship between BPA and GDM has been investigated in some studies, reports on the effects of its analogs (BPS, BPF, and BPAF) on glucose homeostasis of pregnant women are rather limited. To our knowledge, this is the first study to examine the associations between exposures to BPA substitutes and the risk of GDM and plasma glucose levels. In this study, we found that BPAF was associated with an increased risk of GDM and increased plasma glucose levels among pregnant women with normal pre-pregnancy weight. In addition, we observed fetal sex specific effects of BPS on glucose metabolism, which indicated that women carrying a female fetus might be more sensitive and vulnerable to BPS exposure than those carrying a male fetus.

We were aware of five epidemiology studies that had investigated the effects of BPA exposure on glucose metabolism among pregnant women (17–21). Three of them addressed the associations between BPA and GDM, two of which reported null associations and one retrospective study from China reported that urinary BPA levels at the third trimester were associated with a decreased risk of GDM and lower plasma glucose levels (20). Another two studies found positive associations of urinary BPA concentrations with glucose levels during pregnancy. In this study, we did not find associations between BPA and GDM, but we observed non-linear associations between BPA and glucose levels among women who were overweight before pregnancy. The retrospective study from China assessed BPA levels in urine samples just before delivery, while in the present study we used urine samples in early pregnancy, which may contribute to the inconsistence. We observed that moderate BPA exposure were associated with decreased plasma glucose among women who were overweight, which seemed to be opposite to the findings of Bellavia et al.'s (21) study. There are several reasons we assumed this. First, the concentrations of urinary BPA in this study were lower than that reported in Bellavia et al.'s (21) study [geometric means (GM): 0.72 vs. 1.23 for un-adjusted and 0.87 vs. 1.3 for SG-adjusted concentration, ug/L], which may explain the different findings. Second, Bellavia et al. (21) did not exclude women with obesity in their analysis, which may contribute to some confounding effects in the results. Moreover, there are some significant differences in our study population and that of Bellavia et al.'s (21) study, such as races (Chinese vs. American) and the rate of being overweight/obese [18.63% in our study population compared to 44.96% in Bellavia et al.'s (21) study]. Also, Bellavia et al. (21) did not report non-linear associations between BPA exposure and glucose levels. Thus, we believe that our results of BPA and glucose levels among overweight women are not in conflict with Bellavia et al.'s (21), and those could be supplementary to the evidence of disrupting effects of BPA exposure on glucose levels of pregnant women.

The non-monotonic dose-response (NMDR) endocrine-disrupting effects of BPA on glycemia metabolism have been reported in both *in vivo* and *in vitro* studies (9, 40–42). Alonso-Magdalena et al. demonstrated an inverted U-shaped relationship between BPA in environmentally relevant doses and insulin content measured after 48 h (43). Several *in vivo* experiments have reported the great disrupting effects of low-dose BPA on blood glucose homeostasis and pancreatic β-cell function (10, 11, 44, 45). The administration of low-dose BPA exposure (10 μg/kg) among adult mice led to a rise of plasma insulin and induced a rapid decrease in glycemia (44). The U-shaped relationship between BPA and fasting plasma glucose levels found in this study, though only observed among pregnant overweight women, indicates an NMDR effect of BPA exposure on human glucose metabolism, which needs to be verified and investigated in future studies.

Due to the wide and frequent use of BPA substitutes, we also assessed the relationships of BPA substitutes and GDM in this study. We found that urinary BPAF was associated with GDM among women with normal weight. Evidence from cyto-experiments and animal studies suggested that BPAF could be a rather toxic substance (46, 47). Furthermore, our results were generally consistent with a recent case-control study which reported that BPAF and BPS were associated with type 2 diabetes among general population in China (48). We only observed positive associations among women with normal weight, which account for the majority of the participants in this study (63.05%), and the potential explanations might be: (1) overweight women overweight are at a high risk of GDM and the adverse effects of BPAF exposure may be covered; (2) before-pregnancy adiposity status of pregnant women might have interaction effects with BPAF exposure and may lead to different results between women with normal weight and women who are overweight. However, BPAF had the lowest detection rate (42.53%) among the four bisphenols, though we had tried to restrict our analysis in a subgroup of BPAF-detectable women and the results were largely consistent (Table S6). Meanwhile, the number of GDM cases was small (*n* = 16) in the high BPAF category of the overweight group, which may cause a high variance in analysis. Thus, the results should be interpreted with caution and further investigations are required to confirm our findings.

We also found that BPAF and BPS were associated with higher glucose levels among all participants, and fetal sex specific effects were observed for BPS exposure. The disrupting effects on glucose metabolism of BPAF and BPS were reported in animal studies (47). *In vitro* experiments indicated that the potential mechanism of endocrine-disrupting effects of BPAF and BPS might be involved in the stimulation of estrogen receptors (49–51). Consistent with the results of GDM, in the further stratified analysis, we found that BPAF was associated with increased glucose levels among women with normal weight. A possible explanation is that higher adiposity levels imply higher levels of circulating estrogen in women who are overweight, higher circulating estrogen levels could efficiently compete for receptors with BPAF and the disrupting effect of BPAF can be partially eliminated. In this study, we only observed glucose-disrupting effects of BPS among women carrying a female fetus in stratified analysis by fetal sex. We speculate that the fetal sex-difference effects may be attributed to different sex-hormone levels in maternal circulations. Since sex-difference effects of BPS were also reported in another study (52), the underlying mechanism needs to be further studied in detail.

We did not observe any associations between urinary BPF and GDM or glucose levels, though it was reported to be associated with increased 17b-estrodiol (E2), both in animal studies and cell experiments (34, 43). However, it should be noted that BPF was measured in a population with a relatively smaller sample size compared to other bisphenols in this study. However, BPF has the highest detection rate (94.72%) in urine samples of pregnant women, which indicates a ubiquitous exposure to this BPA substitute among the study population. Therefore, more studies with a larger sample size is needed to clarify the potential health effects in human population.

From the aspect of molecular composition, though four bisphenols are similar in chemical structure, a fact that should not be ignored is that BPA and BPF only contains carbon and hydrogen atoms while BPS additionally contains sulfur atom and BPAF additionally contains fluorine atoms which may contribute to different biological effects. A recent animal study also suggested that bisphenols may disrupt the endocrine system in different manners, whereas BPS and BPAF exposure, compared to BPA and BPF exposure, significantly disrupted glucose homeostasis, as reported in this study. Moreover, since BPA was substituted by its analogs, the exposure dose of BPA was lower, while that of BPA substitutes was higher, which may lead to more evident findings in BPAF and BPS (48).

One strength of our study is that we used a cohort-based prospective study design to investigate the associations of urinary bisphenols with GDM and blood glucose levels with adjustment for potential confounders. We also performed a model adjusted to other bisphenols and tested the co-exposure effects. Another strength is that we used the OGTT data of each participant obtained from the medical records system and the diagnosis of GDM was based on the criteria from IADPSG by professional physicians. Moreover, we further analyzed the data stratified by fetal sex and pre-pregnant BMI to investigate the potential modification effects.

In this study, urinary bisphenols levels were used since phenols are mainly excreted into urine, and bisphenols concentrations in urine samples are widely accepted biomarkers of the recent exposures to bisphenols (22, 53, 54). A limitation of this study is that we measured bisphenols in only one spot urine sample for each woman and this may have led to a misclassification of the women's bisphenols exposure. Considering the short biological half-life of bisphenols (BPA < 6 h, BPS < 7 h) (55, 56), one spot urine may be insufficient for an accurate evaluation of BPA exposure. However, according to previous studies, a single spot urine sample is able to predict a subject's tertile categorization which was used for the analyses in this study (57, 58). In addition, we did not collect the information on the source of exposure and we were unable to verify whether human exposure to BPA, BPS, BPF, and BPAF were from the same source. However, this lack of information had no impact on our main results and conclusion of this study. Also, we did not have information on baseline glucose levels and energy intake; thus, it cannot be adjusted in the analysis models. Moreover, the limited sample size in stratified analysis has restricted the power to make a robust conclusion, which should be improved in future studies. Finally, due to the potential differences between women's races, exposure patterns, and random effects, more prospective studies with more accurate exposure evaluations are needed to confirm the findings of our study.

## Conclusion

In this prospective cohort study, we found that BPAF was associated with an increased risk of GDM among pregnant women of normal weight. Additionally, a disrupting effect on plasma glucose was observed for BPS and the effect might be modified by fetal sex. In conclusion, we observed the endocrine-disrupting effects of BPA substitutes (BPS and BPAF) on blood glucose metabolism among Chinese pregnant women, which might constitute potential risk factors of GDM.

## Ethics Statement

This study was approved by the ethics committees of Tongji Medical College, Huazhong University of Science and Technology [No. (2012)07], and Wuhan Maternal and Child Healthcare Hospital (No. 2012003). All participants agreed and signed informed consent.

## Author Contributions

WZ analyzed the data and wrote this manuscript. Valuable suggestions in data analysis and paper writing were gained from WX and SX. WL, XL, and JH helped for urine samples collection and baseline data obtaining. BZ helped a lot in our work conducted in the study hospital. YZ and JL were responsible for urine samples measurements. ZC and YL were in charge of this study and paid a lot of time in revising this manuscript.

### Conflict of Interest Statement

The authors declare that the research was conducted in the absence of any commercial or financial relationships that could be construed as a potential conflict of interest.
