# Supplementary material for: Exposure to Bisphenol a Substitutes and Gestational Diabetes Mellitus: A Prospective Cohort Study in China
Source: Front Endocrinol (Lausanne). 2019 Apr 30;10:262. doi: 10.3389/fendo.2019.00262 (PMC6503732; doi:10.3389/fendo.2019.00262)
Supplement: Supplementary file 1 [file Data_Sheet_1.docx]

| Table S1. Spearman correlation coefficients of urinary bisphenols (n = 776)^a^. | | | | |
| --- | --- | --- | --- | --- |
|  | BPA | BPS | BPAF | BPF |
| BPA | 1.00 |  |  |  |
| BPS | 0.14 | 1.00 |  |  |
| BPAF | 0.27 | 0.20 | 1.00 |  |
| BPF | 0.19 | 0.11 | 0.12 | 1.00 |
| ^a^All the 2-tailed *p* value were < 0.01. | | | | |

| Table S2. Associations between urinary BPAF levels and plasma glucose among different pre-pregnancy BMI group^a^. | | | | | | | | | | | | | | | |
| --- | --- | --- | --- | --- | --- | --- | --- | --- | --- | --- | --- | --- | --- | --- | --- |
| BPAF | GDM | | |  | FPG | |  | 1h-PG | |  | 2h-PG | |  | sum of PG z-score | |
|  | GDM/Total | OR (95% CI) | *p* |  | b (95% CI) | *p* |  | b (95% CI) | *p* |  | b (95% CI) | *p* |  | b (95% CI) | *p* |
| Women with normal weight (n = 1162) | | | | | | | | | | | | | | | |
| per-unit increase in  ln (SG-adj BPAF) | | 1.11 (0.93, 1.33) | 0.25 |  | 0.02 (0.00, 0.05) | 0.02^*^ |  | 0.03 (-0.04, 0.11) | 0.36 |  | 0.03 (-0.03, 0.09) | 0.34 |  | 0.08 (-0.02, 0.18) | 0.13 |
| Low | 46/769 | Reference |  |  | Reference |  |  | Reference |  |  | Reference |  |  | Reference |  |
| High | 38/393 | 1.70 (1.08, 2.67) | 0.02^*^ |  | 0.07 (0.02, 0.12) | < 0.01^*^ |  | 0.10 (-0.08, 0.28) | 0.27 |  | 0.10 (-0.03, 0.24) | 0.14 |  | 0.26 (0.01, 0.50) | 0.04^*^ |
| *p* for trend |  |  | 0.02^*^ |  |  | < 0.01^*^ |  |  | 0.27 |  |  | 0.14 |  |  | 0.04^*^ |
|  |  |  |  |  |  |  |  |  |  |  |  |  |  |  |  |
| Women with overweight (n = 302) | | | | | | | | | | | | | | | |
| per-unit increase in  ln (SG-adj BPAF) | | 0.90 (0.69, 1.19) | 0.46 |  | 0.02 (-0.04, 0.08) | 0.53 |  | 0.01 (-0.16, 0.18) | 0.94 |  | -0.07 (-0.22, 0.08) | 0.38 |  | -0.01 (-0.32, 0.30) | 0.95 |
| Low | 50/223 | Reference |  |  | Reference |  |  | Reference |  |  | Reference |  |  | Reference |  |
| High | 16/79 | 0.97 (0.49, 1.91) | 0.93 |  | 0.11 (-0.05, 0.27) | 0.18 |  | 0.11 (-0.32, 0.55) | 0.61 |  | 0.03 (-0.36, 0.42) | 0.87 |  | 0.33 (-0.46, 1.11) | 0.41 |
| *p* for trend |  |  | 0.93 |  |  | 0.18 |  |  | 0.61 |  |  | 0.87 |  |  | 0.41 |
| Note: SG: specific gravity; FPG: fasting plasma glucose; PG: plasma glucose.  Note: BMI: body mass index; GDM: gestational diabetes mellitus.  ^a^Adjusted for maternal age, educational levels, parity, passive smoking and fetal sex.  ^*^Significant *p* value. | | | | | | | | | | | | | | | |

| Table S3. Associations between urinary BPA levels and plasma glucose among different pre-pregnancy BMI group^a^. | | | | | | | | | | | | | | |  |
| --- | --- | --- | --- | --- | --- | --- | --- | --- | --- | --- | --- | --- | --- | --- | --- |
| BPA | GDM | |  | FPG | |  | 1h-PG | |  | 2h-PG | |  | sum of PG z-score | |  |
|  | GDM/Total | OR (95% CI) |  | β (95% CI) | *p* |  | β (95% CI) | *p* |  | β (95% CI) | *p* |  | β (95% CI) | *p* |  |
| Women with normal weight (n = 1162) | | | | | | | | | | | | | | |  |
| Low | 24/387 | Reference |  | Reference |  |  | Reference |  |  | Reference |  |  | Reference |  |  |
| Medium | 26/381 | 1.14 (0.64, 2.03) |  | -0.02 (-0.08, 0.04) | 0.52 |  | 0.03 (-0.18, 0.23) | 0.81 |  | 0.08 (-0.08, 0.24) | 0.34 |  | 0.04 (-0.25, 0.33) | 0.80 |  |
| High | 34/394 | 1.38 (0.80, 2.39) |  | 0.02 (-0.04, 0.08) | 0.45 |  | -0.09 (-0.29, 0.12) | 0.39 |  | 0.06 (-0.10, 0.22) | 0.46 |  | 0.02 (-0.27, 0.30) | 0.90 |  |
| *p* for trend |  | 0.24 |  |  | 0.31 |  |  | 0.31 |  |  | 0.57 |  |  | 0.94 |  |
|  |  |  |  |  |  |  |  |  |  |  |  |  |  |  |  |
| Women with overweight (n = 302) | | | | | | | | | | | | | | |  |
| Low | 30/103 | Reference |  | Reference |  |  | Reference |  |  | Reference |  |  | Reference |  |  |
| Medium | 18/105 | 0.53 (0.26, 1.06) |  | -0.19 (-0.36, -0.02) | 0.03^*^ |  | -0.34 (-0.79, 0.12) | 0.15 |  | -0.43 (-0.84, -0.03) | 0.03^*^ |  | -0.95 (-1.77, -0.13) | 0.02^*^ |  |
| High | 18/94 | 0.59 (0.29, 1.19) |  | -0.01 (-0.19, 0.16) | 0.88 |  | -0.26 (-0.74, 0.21) | 0.27 |  | -0.41 (-0.83, 0.00) | 0.05 |  | -0.53 (-1.38, 0.32) | 0.22 |  |
| *p* for trend |  | 0.21 |  |  | 0.72 |  |  | 0.40 |  |  | 0.10 |  |  | 0.43 |  |
| Note: SG: specific gravity; FPG: fasting plasma glucose; PG: plasma glucose. | | | | | | | | | | | | | | | |
| Note: BMI: body mass index; GDM: gestational diabetes mellitus. | | | | | | | | | | | | | | | |
| ^a^Adjusted for maternal age, educational levels, parity, passive smoking and fetal sex. | | | | | | | | | | | | | | | |
| ^*^Significant *p* value. | | | | | | | | | | | | | | | |

| Table S4. Associations between urinary BPS levels and plasma glucose among different pre-pregnancy BMI group^a^. | | | | | | | | | | | | |
| --- | --- | --- | --- | --- | --- | --- | --- | --- | --- | --- | --- | --- |
| BPS |  | FPG | |  | 1h-PG | |  | 2h-PG | |  | sum of PG z-score | |
|  |  | β (95% CI) | *p* |  | β (95% CI) | *p* |  | β (95% CI) | *p* |  | β (95% CI) | *p* |
| Women with normal weight (n = 1162) | | | | | | | | | | | | |
| Low |  | Reference |  |  | Reference |  |  | Reference |  |  | Reference |  |
| Medium |  | 0.03 (-0.03, 0.09) | 0.26 |  | 0.16 (-0.05, 0.36) | 0.14 |  | 0.03 (-0.13, 0.19) | 0.71 |  | 0.18 (-0.11, 0.47) | 0.23 |
| High |  | 0.05 (-0.01, 0.11) | 0.09 |  | 0.17 (-0.03, 0.38) | 0.10 |  | 0.09 (-0.08, 0.25) | 0.30 |  | 0.25 (-0.04, 0.54) | 0.09 |
| *p* for trend |  |  | 0.14 |  |  | 0.22 |  |  | 0.30 |  |  | 0.16 |
|  |  |  |  |  |  |  |  |  |  |  |  |  |
| Women with overweight (n = 302) | | | | | | | | | | | | |
| Low |  | Reference |  |  | Reference |  |  | Reference |  |  | Reference |  |
| Medium |  | 0.11 (-0.06, 0.28) | 0.21 |  | 0.24 (-0.22, 0.71) | 0.30 |  | 0.22 (-0.19, 0.63) | 0.30 |  | 0.57 (-0.26, 1.40) | 0.18 |
| High |  | 0.16 (-0.01, 0.33) | 0.07 |  | 0.13 (-0.34, 0.59) | 0.59 |  | 0.02 (-0.39, 0.43) | 0.92 |  | 0.43 (-0.41, 1.26) | 0.31 |
| *p* for trend |  |  | 0.11 |  |  | 0.82 |  |  | 0.80 |  |  | 0.52 |
| Note: SG: specific gravity; FPG: fasting plasma glucose; PG: plasma glucose. | | | | | | | | | | | | |
| Note: BMI: body mass index; GDM: gestational diabetes mellitus. | | | | | | | | | | | | |
| ^a^Adjusted for maternal age, educational levels, parity, passive smoking and fetal sex. | | | | | | | | | | | | |
| ^*^Significant *p* value. | | | | | | | | | | | | |

| Table S5. Associations between urinary BPF levels and plasma glucose among different pre-pregnancy BMI group^a^. | | | | | | | | | | | | |
| --- | --- | --- | --- | --- | --- | --- | --- | --- | --- | --- | --- | --- |
| BPF |  | FPG | |  | 1h-PG | |  | 2h-PG | |  | sum of PG z-score | |
|  |  | β (95% CI) | *p* |  | β (95% CI) | *p* |  | β (95% CI) | *p* |  | β (95% CI) | *p* |
| Women with normal weight (n = 479) | | | | | | | | | | | | |
| Low |  | Reference |  |  | Reference |  |  | Reference |  |  | Reference |  |
| Medium |  | 0.05 (-0.04, 0.14) | 0.29 |  | 0.08 (-0.24, 0.41) | 0.62 |  | 0.01 (-0.23, 0.25) | 0.93 |  | 0.09 (-0.32, 0.50) | 0.67 |
| High |  | 0.06 (-0.03, 0.15) | 0.21 |  | 0.04 (-0.29, 0.36) | 0.82 |  | -0.10 (-0.34, 0.14) | 0.41 |  | 0.05 (-0.36, 0.46) | 0.80 |
| *p* for trend |  |  | 0.35 |  |  | 0.99 |  |  | 0.32 |  |  | 0.94 |
|  |  |  |  |  |  |  |  |  |  |  |  |  |
| Women with overweight (n = 143) | | | | | | | | | | | | |
| Low |  | Reference |  |  | Reference |  |  | Reference |  |  | Reference |  |
| Medium |  | -0.14 (-0.47, 0.20) | 0.42 |  | -0.34 (-1.17, 0.48) | 0.42 |  | -0.76 (-1.52, -0.00) | 0.05 |  | -0.99 (-2.51, 0.53) | 0.20 |
| High |  | -0.12 (-0.43, 0.19) | 0.44 |  | -0.38 (-1.15, 0.38) | 0.32 |  | -0.58 (-1.28, 0.12) | 0.10 |  | -0.88 (-2.27, 0.52) | 0.22 |
| *p* for trend |  |  | 0.67 |  |  | 0.49 |  |  | 0.43 |  |  | 0.47 |
| Note: SG: specific gravity; FPG: fasting plasma glucose; PG: plasma glucose. | | | | | | | | | | | | |
| Note: BMI: body mass index; GDM: gestational diabetes mellitus. | | | | | | | | | | | | |
| ^a^Adjusted for maternal age, educational levels, parity, passive smoking and fetal sex. | | | | | | | | | | | | |
| ^*^Significant *p* value. | | | | | | | | | | | | |

| Table S6. Sensitivity analysis for BPAF by excluding women with undetectable urinary BPAF concentrations among women with normal weight (n = 517). | | | | | | | | |
| --- | --- | --- | --- | --- | --- | --- | --- | --- |
| BPAF |  | GDM/Total |  | GDM |  |  | FPG |  |
|  |  |  |  | Model 1 | Model 2 |  | Model 1 | Model 2 |
|  |  |  |  | OR (95% CI) | OR (95% CI) |  | β (95% CI) | β (95% CI) |
|  |  | 49/517 |  |  |  |  |  |  |
| Low |  | 20/261 |  | reference | reference |  | reference | reference |
| High |  | 29/256 |  | 1.53 (0.83, 2.83) | 1.44 (0.76, 2.73) |  | 0.08 (0.01, 0.16)^*^ | 0.08 (0.00, 0.16)^*^ |
| Note: BMI: body mass index; GDM: gestational diabetes mellitus. | | | | | | | | |
| Model 1: Adjusted for maternal age, educational levels, parity, passive smoking and fetal sex. | | | | | | | | |
| Model 2: Additonally adjusted for other bisphenols based on model 1. | | | | | | | | |
| ^*^Significant *p* value. | | | | | | | | |

| 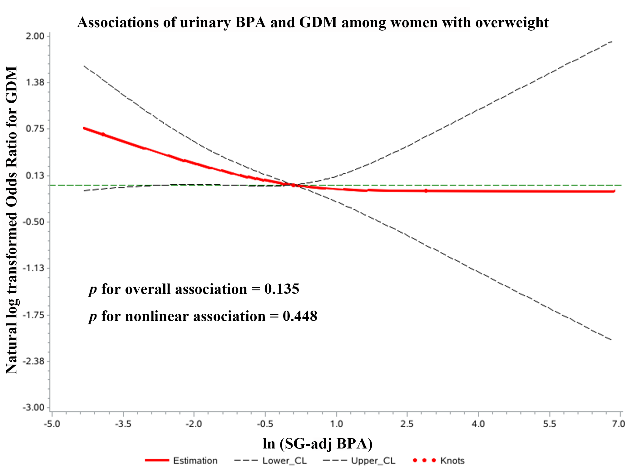 |
| --- |
| Figure S1. Restricted cubic spline for the associations between urinary BPA and plasma glucose levels among women with overweight. The red lines represent differences in glucose levels for natural log transformed specific gravity adjusted urinary BPA concentration with adjustment for maternal age, educational levels, parity, passive smoking and fetal sex. Knots were set at the 5^th^, 50^th^, 95^th^ percentiles and the reference value was set to median of urinary BPA distribution among women with overweight. Dashed lines represent 95% CI. |

| Table S7. Associations of urinary bisphenols and GDM or glucose levels, stratified by fetal sex. | | | | | | | | | |
| --- | --- | --- | --- | --- | --- | --- | --- | --- | --- |
| Bisphenols |  |  | Male |  |  |  | Female |  |  |
| GDM |  |  | OR^a^ or β^a^ | 95% CI | |  | OR^a^ or β^a^ | 95% CI | |
| BPA |  |  |  |  |  |  |  |  |  |
| Low |  |  | Reference | | |  | Reference | | |
| Medium | |  | 0.91 | 0.51 | 1.64 |  | 0.71 | 0.40 | 1.27 |
| High |  |  | 0.93 | 0.51 | 1.69 |  | 0.85 | 0.47 | 1.52 |
|  |  |  |  |  |  |  |  |  |  |
| BPS |  |  |  |  |  |  |  |  |  |
| Low |  |  | Reference | | |  | Reference | | |
| Medium | |  | 1.06 | 0.60 | 1.87 |  | 1.10 | 0.62 | 1.95 |
| High |  |  | 0.70 | 0.38 | 1.30 |  | 1.00 | 0.55 | 1.81 |
|  |  |  |  |  |  |  |  |  |  |
| BPAF |  |  |  |  |  |  |  |  |  |
| Low |  |  | Reference | | |  | Reference | | |
| High |  |  | 1.18 | 0.68 | 2.02 |  | 1.39 | 0.82 | 2.37 |
|  |  |  |  |  |  |  |  |  |  |
| FPG |  |  |  |  |  |  |  |  |  |
| BPA |  |  |  |  |  |  |  |  |  |
| Low |  |  | Reference | | |  | Reference | | |
| Medium | |  | -0.07 | -0.14 | 0.00 |  | -0.02 | -0.10 | 0.06 |
| High |  |  | -0.01 | -0.09 | 0.06 |  | 0.01 | -0.07 | 0.09 |
|  |  |  |  |  |  |  |  |  |  |
| BPS |  |  |  |  |  |  |  |  |  |
| Low |  |  | Reference | | |  | Reference | | |
| Medium | |  | 0.03 | -0.04 | 0.10 |  | 0.04 | -0.04 | 0.12 |
| High |  |  | -0.01 | -0.08 | 0.06 |  | 0.10* | 0.02* | 0.18* |
|  |  |  |  |  |  |  |  |  |  |
| BPAF |  |  |  |  |  |  |  |  |  |
| Low |  |  | Reference | | |  | Reference | | |
| High |  |  | 0.05 | -0.01 | 0.11 |  | 0.03 | -0.05 | 0.10 |
|  |  |  |  |  |  |  |  |  |  |
| 1h-PG |  |  |  |  |  |  |  |  |  |
| BPA |  |  |  |  |  |  |  |  |  |
| Low |  |  | Reference | | |  | Reference | | |
| Medium | |  | 0.05 | -0.18 | 0.29 |  | -0.21 | -0.45 | 0.03 |
| High |  |  | 0.00 | -0.24 | 0.25 |  | -0.18 | -0.43 | 0.07 |
|  |  |  |  |  |  |  |  |  |  |
| BPS |  |  |  |  |  |  |  |  |  |
| Low |  |  | Reference | | |  | Reference | | |
| Medium | |  | 0.20 | -0.04 | 0.43 |  | 0.07 | -0.17 | 0.31 |
| High |  |  | 0.01 | -0.23 | 0.26 |  | 0.31* | 0.06* | 0.56* |
|  |  |  |  |  |  |  |  |  |  |
| BPAF |  |  |  |  |  |  |  |  |  |
| Low |  |  | Reference | | |  | Reference | | |
| High |  |  | 0.05 | -0.16 | 0.27 |  | -0.02 | -0.24 | 0.21 |
|  |  |  |  |  |  |  |  |  |  |
| 2h-PG |  |  |  |  |  |  |  |  |  |
| BPA |  |  |  |  |  |  |  |  |  |
| Low |  |  | Reference | | |  | Reference | | |
| Medium | |  | 0.07 | -0.11 | 0.26 |  | -0.11 | -0.32 | 0.09 |
| High |  |  | 0.07 | -0.12 | 0.26 |  | -0.12 | -0.34 | 0.09 |
|  |  |  |  |  |  |  |  |  |  |
| BPS |  |  |  |  |  |  |  |  |  |
| Low |  |  | Reference | | |  | Reference | | |
| Medium | |  | -0.04 | -0.23 | 0.15 |  | 0.00 | -0.20 | 0.21 |
| High |  |  | -0.07 | -0.26 | 0.12 |  | 0.09 | -0.12 | 0.30 |
|  |  |  |  |  |  |  |  |  |  |
| BPAF |  |  |  |  |  |  |  |  |  |
| Low |  |  | Reference | | |  | Reference | | |
| High |  |  | 0.07 | -0.10 | 0.24 |  | 0.02 | -0.18 | 0.21 |
|  |  |  |  |  |  |  |  |  |  |
| sum of PG z-score |  |  |  |  |  |  |  |  |  |
| BPA |  |  |  |  |  |  |  |  |  |
| Low |  |  | Reference | | |  | Reference | | |
| Medium | |  | -0.05 | -0.38 | 0.29 |  | -0.27 | -0.66 | 0.12 |
| High |  |  | 0.01 | -0.34 | 0.36 |  | -0.19 | -0.59 | 0.21 |
|  |  |  |  |  |  |  |  |  |  |
| BPS |  |  |  |  |  |  |  |  |  |
| Low |  |  | Reference | | |  | Reference | | |
| Medium | |  | 0.14 | -0.20 | 0.48 |  | 0.12 | -0.27 | 0.51 |
| High |  |  | -0.09 | -0.44 | 0.26 |  | 0.47* | 0.07* | 0.86* |
|  |  |  |  |  |  |  |  |  |  |
| BPAF |  |  |  |  |  |  |  |  |  |
| Low |  |  | Reference | | |  | Reference | | |
| High |  |  | 0.17 | -0.14 | 0.48 |  | 0.05 | -0.31 | 0.41 |

Note: GDM: gestational diabetes mellitus; PG: plasma glucose.

^a^: adjusted for other bisphenols and maternal age, educational levels, parity, passive smoking.

*: significant with *p* < 0.05.
